# A Comparative Analysis of the Impact of Severe Acute Respiratory Syndrome Coronavirus 2 Infection on the Performance of Clinical Decision-Making Algorithms for Pulmonary Embolism

**DOI:** 10.3390/jcm13237008

**Published:** 2024-11-21

**Authors:** Merve Eksioglu, Burcu Azapoglu Kaymak, Atilla Halil Elhan, Tuba Cimilli Ozturk

**Affiliations:** 1 Department of Emergency Medicine, Fatih Sultan Mehmet Education and Research Hospital, University of Health Sciences, Istanbul 34752, Turkey; burcuazapkaymak@gmail.com (B.A.K.); tcimilliozturk@gmail.com (T.C.O.); 2 Department of Biostatistics, Ankara University Faculty of Medicine, Ankara 06230, Turkey; elhan@medicine.ankara.edu.tr

**Keywords:** coronavirus, SARS-CoV-2 infection, computed tomography pulmonary angiography, pulmonary embolism, D-dimer, Wells score, Geneva score, YEARS algorithm, PEGeD algorithm

## Abstract

**Background/Objectives**: This study aimed to compare the diagnostic accuracy of the Wells and Geneva scores using a 500 ng/mL D-dimer cutoff, as well as the age-adjusted D-dimer (AADD), YEARS, and pulmonary embolism graduated D-dimer (PEGeD) algorithms, in patients with and without COVID-19. Various D-dimer cutoffs were also evaluated. **Methods:** This retrospective study included emergency department patients who underwent computed tomography pulmonary angiography (CTPA) for suspected pulmonary embolism (PE). The diagnostic performances of clinical prediction algorithms were compared between COVID-19-positive and -negative groups. **Results:** We analyzed data from 1423 patients; the PE and COVID-19 positivity rates were 7.3% and 69.9%, respectively. In COVID-19-positive patients, the Wells score with a 500 ng/mL D-dimer cutoff demonstrated 97.22% sensitivity (95% CI: 80.53–100.00) and 4.99% specificity (95% CI: 3.58–6.39). Using AADD raised the specificity to 7.81% (95% CI: 6.08–9.54) while maintaining 97.22% sensitivity (95% CI: 93.43–100.00); similar findings were observed with the Geneva score. The YEARS algorithm had 86.11% sensitivity (95% CI: 78.12–94.10) and 32.75% specificity (95% CI: 29.73–35.78), whereas the PEGeD algorithm showed 86.11% sensitivity (95% CI: 78.12–94.10) and 34.06% specificity (95% CI: 31.00–37.12). Both algorithms demonstrated slightly improved specificity and accuracy in COVID-19-positive patients. **Conclusions:** The YEARS and PEGeD algorithms showed slight improvements in specificity and accuracy among COVID-19-positive patients. The Wells and Geneva scores maintained higher sensitivity but lower specificity across groups. Adjusting the D-dimer cutoffs increased the specificity but increased the risk of missed diagnoses. Overall, COVID-19 had a minimal impact on PE diagnostic algorithm performances.

## 1. Introduction

Pulmonary embolism (PE) is a serious complication of coronavirus disease 2019 (COVID-19) [1,2,3]. The strategies for diagnosing PE in the emergency department are based on several clinically validated algorithms developed to safely limit the use of radiation-based imaging modalities, specifically computed tomography pulmonary angiography (CTPA), which is considered the gold standard [4]. These clinical algorithms combine the pretest probability with D-dimer results to classify the risk and guide the indication for CTPA. The Wells and Geneva scores, used in conjunction with D-dimer measurements, are widely used in PE diagnosis and are the preferred tools for assessing the pretest clinical probability [5,6]. These scores determine the PE likelihood upon the assessment of symptoms, medical history, and risk factors, as well as provide guidance for excluding PE or determining the need for further diagnostic testing when used in combination with D-dimer testing. New algorithms, such as age-adjusted D-dimer (AADD), YEARS, and pulmonary embolism graduated D-dimer (PEGeD), have been introduced to safely limit CTPA use [7,8,9].

Although it is well established that COVID-19 increases the risk of venous thromboembolism (VTE) in the general population, it is unclear whether this increase poses a higher risk in emergency department patients with suspected PE [10,11]. Because PE and COVID-19 have similar signs and symptoms, this led to significant diagnostic challenges for emergency physicians during the COVID-19 pandemic [12]. The hyperinflammatory state and higher D-dimer levels in patients with COVID-19 made it difficult to rule out PE without CTPA, leading to increased CTPA requests [13,14,15,16]. Moreover, several studies have suggested that the clinical prediction models used to determine the probability of PE in the general population are not fully applicable to patients with COVID-19 [17,18,19]. In this context, echocardiography, especially with advanced techniques such as speckle-tracking, may provide complementary diagnostic support by identifying subtle cardiac strain abnormalities associated with COVID-19, potentially aiding in PE risk assessment in borderline cases [20].

In this study, we primarily aimed to comprehensively investigate the diagnostic performance of several clinical algorithms for PE diagnosis during the COVID-19 pandemic. Moreover, we aimed to compare the effectiveness of these algorithms in patient groups that tested positive and negative for COVID-19. We also analyzed the diagnostic performances of different D-dimer cutoffs used in PE prediction.

## 2. Materials and Methods

The Ethics Committee of Health Sciences University Fatih Sultan Mehmet Training and Research Hospital approved this retrospective cohort study (Ethics Approval Protocol Number: 2023/112; Approval Date: 12 October 2023). This study included patients aged ≥ 18 years who presented to the emergency department of Fatih Sultan Mehmet Training and Research Hospital between 1 May 2020, and 1 June 2021, and underwent CTPA for suspected PE. We excluded patients with incomplete or inadequate medical records, defined as missing essential documentation on symptoms, medical history, or physical findings required for calculating pretest scores. Patients were also excluded if they did not have a D-dimer test result, had inconclusive CTPA results, or met any of the following additional criteria: known pregnancy, genetic thrombotic disorders, or therapeutic anticoagulant use for indications other than VTE or suspected VTE. Inconclusive CTPA was defined as imaging studies where the results were indeterminate for the presence or absence of PE, often due to poor image quality or technical limitations.

We collected data on patient backgrounds, demographics, clinical findings, D-dimer levels, and risk factors for PE (immobilization or a history of surgery in the last month, a history of previous deep vein thrombosis [DVT] or PE, malignancy, hemoptysis, symptoms and findings of DVT, CTPA findings, and coronavirus disease 2019 (COVID-19) reverse transcription polymerase chain reaction [RT-PCR] test results). As this was a retrospective study, informed consent was not required. An investigator blinded to the CTPA reports collected demographic, clinical, and laboratory data.

### 2.1. Scores and Evaluation of Algorithm

We retrospectively calculated the components of diagnostic prediction rules based on the clinical data records at the time of the CTPA request. We excluded components for any undocumented score.

We identified the item “PE is the most likely diagnosis/alternate diagnosis is less likely” in the patient’s medical record based on the following criteria:

- The physician stated that PE was the most likely diagnosis in the medical record; or
- No other YEARS criteria were specified in the medical record but a CTPA procedure was performed when the D-dimer level was <1000 μg/L within 24 h of D-dimer measurement.

We rated this subjective item as 0 if the criteria were not met [21].

The Wells score ranges from 0 to 12.5 points and is calculated based on certain criteria: signs and symptoms of DVT, PE as the most likely diagnosis, previous PE/DVT diagnosis, heart rate >100 beats/min, immobilization or recent surgery, malignancy, and hemoptysis [9,22]. Accordingly, based on the Wells score, we herein categorized patients into low- (<4 points), moderate- (4.5–6 points), and high- (≥6.5 points) risk groups [23].

The revised Geneva score considers certain factors, including previously diagnosed PE/DVT, unilateral lower extremity pain, heart rate, active malignancy, hemoptysis, age > 65 years, and pain on extremity palpation, when assessing the risk for PE [9]. Based on this score, we categorized patients into low- (0–3 points), moderate- (4–10 points), and high- (≥11 points) risk groups [23].

In the standard protocol, patients at high risk of PE as per the Wells or Geneva scores are evaluated with CTPA, and patients at low and moderate risk are evaluated with CTPA when D-dimer levels are above 500 ng/mL or above age-adjusted cutoff values. The AADD cutoff was defined as 10 times the age in patients older than 50 years [24,25].

The YEARS algorithm assesses PE by considering D-dimer levels alongside criteria such as hemoptysis, signs of DVT, and PE being the most likely diagnosis. PE is excluded in patients with D-dimer levels below 1000 ng/mL and those meeting zero YEARS criteria, whereas CTPA is required in patients who meet one or more YEARS criteria and have D-dimer levels above 500 ng/mL [7,26].

The PEGeD algorithm excludes PE in patients with a low pretest probability and a D-dimer level below 1000 ng/mL or an intermediate pretest probability and a D-dimer level below 500 ng/mL. All other patients, including those in the high-risk group, are evaluated using CTPA [9,27]. In this algorithm, the pretest clinical probability is determined based on the Wells score.

### 2.2. COVID-19 Assessment

We evaluated the COVID-19 status based on RT-PCR test results obtained within 30 days prior to imaging. Patients were classified as COVID-19-positive if they had a positive RT-PCR test result at the time of their emergency department visit or within the preceding 30 days. Additionally, patients presenting with clinical symptoms of COVID-19, receiving COVID-19 treatment, and showing typical viral pneumonia findings upon CTPA imaging—per the criteria from the Radiological Society of North America for COVID-19 CT findings, which include characteristic peripheral bilateral ground-glass opacities with or without consolidation, visible intralobular lines, or “crazy-paving” patterns—were also classified as COVID-19-positive [28]. This comprehensive approach helped us identify COVID-19 cases through a combination of clinical, radiological, and laboratory criteria rather than relying solely on RT-PCR results.

### 2.3. CTPA Protocol

CTPA was performed by administering an intravenous 50–55 mL injection of iodinated contrast medium, followed by the procedure using a 128-slice multidetector CT scanner (GE Healthcare Goldseal Optima CT 660). We diagnosed PE based on the presence of filling defects in at least two consecutive axial sections of the pulmonary artery. An emergency room physician who was blinded to the clinical information reviewed the CTPA scans of the patients included in the study. The CTPA results were recorded as positive or negative for PE.

### 2.4. Statistical Analysis

The descriptive statistics were presented as counts and percentages for categorical variables and as the median (interquartile range) for non-normally distributed continuous variables or ordinal variables. The normality of continuous variables was assessed using the Shapiro–Wilk test. Intergroup differences for categorical variables were evaluated using the chi-squared test or Fisher’s exact test, as appropriate. Differences between two groups in continuous variables that did not meet the normality assumption, as well as ordinal variables, were analyzed using the Mann–Whitney U test. Receiver operating characteristic (ROC) curves were used to assess the diagnostic value of alternative tests, with the area under the curve (AUC) calculated following the method used by Hanley and McNeil [29]. An area under the ROC curve of 0.5 indicates that the discriminatory ability of an alternative test is no better than chance. Additionally, the sensitivity, specificity, positive and negative predictive values (PPV and NPV), likelihood ratios (LR), and accuracy were calculated with 95% confidence intervals. A *p*-value of less than 0.05 indicated statistical significance.

## 3. Results

**Figure 1:**
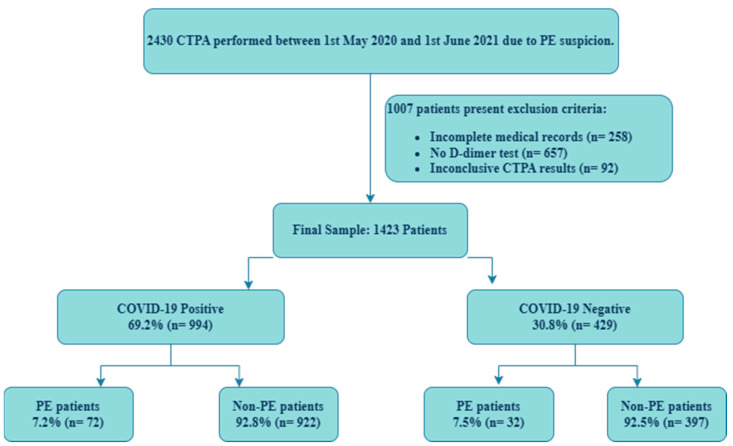
Study flowchart. Abbreviations: CTPA, computed tomography pulmonary angiography; PE, pulmonary embolism. Note: “Incomplete medical records” indicates cases missing key documentation on symptoms, history, or findings, which are needed for calculating pretest scores.

In this study, we analyzed data from 2430 patients who underwent CTPA for suspected PE between 1 May 2020 and 1 June 2021. We excluded 1007 patients because of missing data and inconclusive examinations and included the remaining 1423 patients in the analyses (Figure 1).

### 3.1. Patient Demographics

The median age of the patients was 63.0 years (IQR: 49.0–76.0). Regarding the distribution by sex, 54.7% of the patients were female (779 patients) and 45.3% were male (644 patients). The COVID-19 status was positive in 69.2% of patients (994 patients) and negative in 30.8% (429 patients). Among those classified as COVID-19-positive, 25.8% (256 patients) had a negative PCR result but were considered positive based on consistent clinical and imaging findings. Overall, 7.3% (104 patients) were diagnosed with PE, with no significant difference in PE rates between COVID-19-positive and -negative patients (*p* = 0.886). Among patients without COVID-19, there were higher rates of clinical signs of DVT (6.8% vs. 2.9%, *p* = 0.001) and significantly elevated Geneva scores (median (IQR): 5.0 [4.0–6.0] vs. 5.0 [3.0–6.0], *p* = 0.003). Furthermore, these patients were more frequently classified as having a moderate-to-high probability on both the Wells and Geneva scores, suggesting that COVID-19-negative patients for whom CTPA was indicated presented with higher assessed pretest probabilities of PE (Table 1).

There was a significant difference in the median Wells scores between patients who were and were not diagnosed with PE; the former group had a median Wells score of 1.5 (IQR: 0.0–3.0) and the latter had a score of 1.0 (IQR: 0.0–1.5) (*p* < 0.001). Similarly, patients diagnosed with PE had a median Geneva score of 6.0 (IQR: 4.0–8.0), whereas patients not diagnosed with PE had a score of 5.0 (IQR: 3.0–6.0); this difference was statistically significant (*p* < 0.001) (Table S1). Additionally, certain factors, including age > 65 years, a previous history of DVT/PE, clinical DVT findings, a history of surgery or fracture in the last 1 month, immobilization, unilateral leg edema, and unilateral leg pain, were significantly more prevalent in patients diagnosed with PE (Table 2). However, we found no Intergroup difference in terms of malignancy, heart rate > 100 bpm, and hemoptysis rates.

### 3.2. Score and Algorithms

#### 3.2.1. Diagnostic Performance of Wells and Geneva Scores

The cutoff value of 500 ng/mL D-dimer for the Wells score exhibited 96.15% (95% CI: 92.46–99.85%) sensitivity and 5.16% (95% CI: 3.96–6.35%) specificity, with an AUC of 0.507 (95% CI: 0.480–0.533). The Wells score with AADD exhibited 95.19% (95% CI: 91.08–99.30%) sensitivity, 8.11% (95% CI: 6.64–9.59%) specificity, and 0.517 (95% CI: 0.490–0.543) AUC. The Geneva score with 500 ng/mL D-dimer had a 96.15% (95% CI: 92.46–99.85%) sensitivity, 5.08% (95% CI: 3.89–6.26%) specificity, and 0.506 (95% CI: 0.480–0.532) AUC, whereas the Geneva score with AADD had a 95.19% (95% CI: 91.08–99.30%) sensitivity, 8.04% (95% CI: 6.57–9.50%) specificity, and 0.516 (95% CI: 0.490–0.542) AUC (Table S2). We found no significant differences in sensitivity and specificity between the Wells score and Geneva algorithm (*p* > 0.05).

#### 3.2.2. Diagnostic Performance of YEARS and PEGeD Algorithms

The YEARS algorithm had a sensitivity of 85.58% (95% CI: 78.82–92.33%), a specificity of 30.93% (95% CI: 28.44–33.43%), and an AUC of 0.583 (95% CI: 0.556–0.608). The PEGeD algorithm had a sensitivity of 85.58% (95% CI: 78.82–92.33%), a specificity of 32.22% (95% CI: 29.70–34.74%), and an AUC of 0.589 (95% CI: 0.563–0.615) (Table S2). The Wells and Geneva scores showed higher sensitivity than YEARS and PEGeD (*p* < 0.001) but the YEARS and PEGeD algorithms were superior in terms of specificity (*p* < 0.0001).

#### 3.2.3. Performance of Algorithms by COVID-19 Status

**Figure 2:**
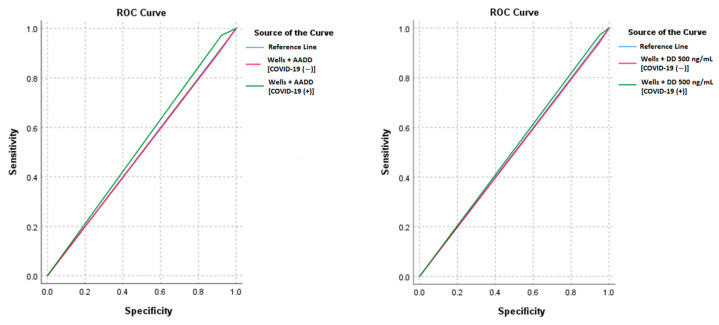
Receiver operating characteristic curves for Wells + age-adjusted D-dimer and Wells + D-dimer 500 ng/mL cutoffs in COVID-19-positive and -negative patients for predicting pulmonary embolism.

**Figure 3:**
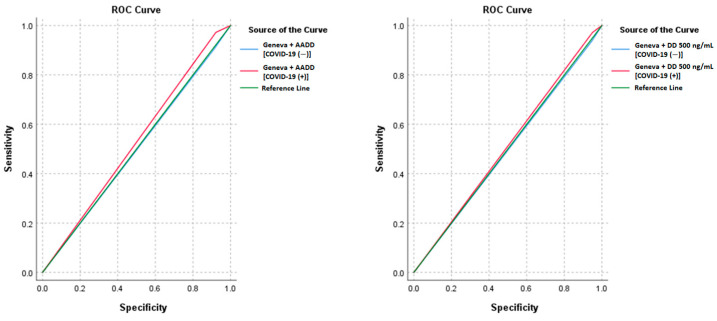
Receiver operating characteristic curves for Geneva scores combined with age-adjusted D-dimer and Geneva scores with a D-dimer cutoff of 500 ng/mL in COVID-19-positive and -negative patients for predicting pulmonary embolism.

With the Wells score and 500 ng/mL D-dimer cutoff, there was a sensitivity of 93.75% and a specificity of 5.54% in patients without COVID-19 and a sensitivity of 97.22% and a specificity of 4.99% in patients with COVID-19. With AADD, these rates were 90.63% for sensitivity and 8.82% for specificity in patients without COVID-19 and 97.22% for sensitivity and 7.81% for specificity in patients with COVID-19. Similar to the Geneva score, there was a sensitivity of 93.75% and a specificity of 5.29% in patients without COVID-19 and a sensitivity of 97.22% and a specificity of 4.99% in patients with COVID-19. With AADD, there was a sensitivity of 90.63% and a specificity of 8.56% in patients without COVID-19 and a sensitivity of 97.22% and a specificity of 7.81% in patients with COVID-19. There was no significant difference between COVID-19-positive and -negative patients in terms of sensitivity, specificity, PPV, NPV, or accuracy for the Wells and Geneva scores using both the 500 ng/mL D-dimer cutoff and AADD approach (*p* > 0.05) (Table 3). Additionally, the AUC values did not significantly differ between the COVID-19 groups (*p* > 0.05), suggesting that the diagnostic performance of these algorithms is consistent across COVID-19 statuses (Figure 2 and Figure 3).

**Figure 4:**
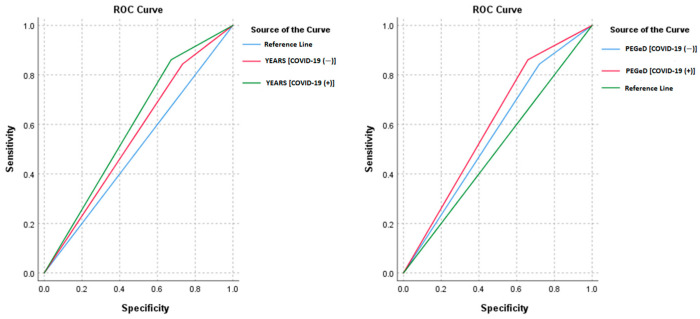
Receiver operating characteristic curves for the diagnostic performance of the YEARS algorithm (**left**) and PEGeD algorithm (**right**) in predicting pulmonary embolism among COVID-19-positive and -negative patient groups.

With the YEARS algorithm, there was a sensitivity of 84.38% and a specificity of 26.70% in patients without COVID-19 and a sensitivity of 86.11% and a specificity of 32.75% in patients with COVID-19. The accuracy rates were 31.00% in patients without COVID-19 and 36.62% in patients with COVID-19. There were significant differences in specificity (*p* = 0.029) and accuracy (*p* = 0.041) between patients with and without COVID-19 (Table 3). The PEGeD algorithm had a sensitivity of 84.38% and specificity of 27.96% in patients without COVID-19 and a sensitivity of 86.11% and a specificity of 34.06% in patients with COVID-19. The accuracy rates in patients without and with COVID-19 were 32.17% and 37.83%, respectively. There were also significant differences in terms of the PEGeD algorithm by specificity (*p* = 0.030) and accuracy (*p* = 0.041) (Table 4). The AUC values were slightly higher for both the YEARS (0.594 [0.533–0.656]) and PEGeD (0.601 [0.540–0.662]) algorithms in COVID-19-positive patients than in COVID-19-negative patients (YEARS: 0.555 [0.458–0.653]; PEGeD: 0.562 [0.465–0.659]). However, these AUC differences were not statistically significant (*p* > 0.05), indicating similar overall diagnostic performance across COVID-19-positive and -negative groups (Figure 4).

### 3.3. Diagnostic Performance of D-Dimer Cutoff Values

In the present study, we reviewed the diagnostic performance of different cutoff values based on patients’ D-dimer levels. The 500-ng/mL cutoff value had a sensitivity of 96.15%, a specificity of 5.08%, and an NPV of 94.37%. With this cutoff value, 67 unnecessary CTPAs were avoided but 4 PE diagnoses were missed. At a cutoff value of 1000 ng/mL, the sensitivity decreased to 81.73%, the specificity increased to 33.13%, and the NPV was 95.83%; accordingly, 437 CTPAs were avoided and 19 PE diagnoses were missed. A cutoff value of 2390 ng/mL reduced the sensitivity to 52.88% and increased the specificity to 73.77%; therefore, at this level, 973 CTPAs were avoided and 49 PE diagnoses were missed (Table 5).

## 4. Discussion

In this study, we compared the diagnostic performance of the most commonly used clinical decision-making algorithms for PE diagnosis during the COVID-19 pandemic. Our results indicated that all algorithms exhibited similar specificity and sensitivity to those of the prepandemic period. The algorithms that used Wells and Geneva scores with D-dimer cutoff values performed similarly in patients with and without COVID-19. The YEARS and PEGeD algorithms performed significantly better in patients with COVID-19 than in patients without COVID-19 in terms of specificity and accuracy. The Wells and Geneva scores provided higher sensitivity when used with different D-dimer cutoffs but low specificity limited the diagnostic performance of these algorithms. Higher D-dimer cutoffs increased the specificity; however, the decrease in sensitivity increased the risk of missed PE cases.

The results of the PEPCOV international retrospective study indicated that COVID-19 did not increase the likelihood of PE diagnosis in the emergency department [30]. The PEPCOV study reviewed the PE diagnosis rates between patients with and without COVID-19, and a multivariate logistic regression analysis revealed that COVID-19 was not associated with an increased risk of PE and that there was no significant effect of COVID-19 on PE risk (adjusted OR = 0.98; 95% CI = 0.76–1.26). This result also applied to patients who underwent CTPA, particularly during the pandemic, indicating that COVID-19 did not increase the risk of PE. We found no significant difference in the rates of PE diagnosis between patients with and without COVID-19 in the present study, which is consistent with the results of the PEPCOV study.

In the present study, the area under the ROC (AUROC) values for clinical decision-making algorithms used for PE diagnosis remained consistently low in both COVID-19-positive and -negative groups, indicating a limited ability of these algorithms to effectively differentiate between patients with and without PE. This finding aligns with previous studies, such as that by Kirsch et al., who reported an AUROC of 0.54 for the Wells score in COVID-19 patients, highlighting its limited diagnostic accuracy in this context [31]. Similarly, a study conducted in hospitalized COVID-19 patients found that the modified Wells score demonstrated restricted utility, with an AUROC of 0.611, underscoring its inadequate performance in diagnosing PE within this population [32]. Silva et al. also observed AUROC values as low as 0.520 when comparing traditional clinical scores with the AADD, YEARS, and PEGeD algorithms, further emphasizing the limited discriminative capacity of these tools in COVID-19 settings [19]. In a separate study that excluded COVID-19 patients, Silva et al. reported that the AADD strategy improved the specificity among patients older than 70 years and safely reduced CTPA requests without significantly lowering the sensitivity, whereas the YEARS and PEGeD algorithms exhibited higher specificity across all age groups [33]. Our study builds on these findings by demonstrating that these low AUROC values persist in COVID-19-negative patients, suggesting that the limited performance of these algorithms are an inherent limitation rather than one exclusive to patients with COVID-19. This limitation may partly arise from subjective elements within the scoring systems, such as the Wells score component “Is PE the most likely diagnosis?”, which introduces variability and can compromise the predictive accuracy. In emergency department settings, where COVID-19 symptoms such as hypoxemia and tachycardia overlap with those of PE, clinicians may tend to overestimate the PE risk, further diminishing the specificity of these scores. Additionally, the selective inclusion of patients who underwent CTPA for suspected PE in our study likely contributed to the low AUROC values observed. By focusing on a cohort with high pretest probability, the discriminative power of these algorithms may be reduced, as these clinical scores were initially intended to identify low-risk patients who could safely avoid imaging. This limitation has been previously noted as a challenge for accurate PE prediction, particularly within high-risk emergency department cohorts. Our findings underscore the need to refine PE diagnostic algorithms to improve the predictive accuracy in high-risk settings, such as emergency departments, and across diverse patient populations. Future studies should aim to develop and validate new, integrated models that address these limitations, enabling safer and more effective PE diagnoses in both COVID-19 and non-COVID-19 patients.

In a study by Chassagnon et al., it was suggested that PE exclusion strategies in patients with COVID-19 should not differ from those in patients without COVID-19, but the D-dimer level was the most important predictor [34]. The results of the present study indicated that clinical algorithms based on Wells and Geneva scores provided higher sensitivity but lower specificity and limited discriminative capacity in patients with COVID-19, which was consistent with the results of previous studies [19,34,35]. Another study that investigated the combined use of the Wells score and D-dimer levels in the diagnosis of DVT and PE in patients with COVID-19 similarly reported that the combination of the Wells PE score and D-dimer levels provided higher sensitivity but lower specificity [35]. The study reported that COVID-19-specific factors (e.g., intubation and severe systemic inflammation) were important predictors of PE occurrence and that PE may be usually asymptomatic and not significantly associated with classical risk factors. Conversely, in the present study, classical thromboembolic risk factors were more prevalent in patients diagnosed with PE. The relatively larger population in the present study and the inclusion of patients without COVID-19, along with those with COVID-19, may explain this difference. The present study provides a comprehensive perspective on PE diagnosis in patients with and without COVID-19, demonstrating the need to reassess the importance of classical risk factors.

International guidelines recommend the use of the YEARS and PEGeD algorithms as effective alternatives to standard methods for PE diagnosis and management [23]. These algorithms are designed to reduce unnecessary CTPA requests by adjusting D-dimer cutoffs based on the clinical context, enhancing the specificity without substantially compromising the sensitivity. Studies have demonstrated that the YEARS and PEGeD algorithms effectively balance the diagnostic accuracy with the goal of minimizing imaging use. For instance, a recent study by Vielhauer et al. highlighted the superior performance of the PEGeD and YEARS algorithms in a COVID-19 cohort, achieving high sensitivity (95.7% and 95.6%, respectively) while reducing the need for diagnostic imaging by 14–15% [36]. The improved performance of the YEARS and PEGeD algorithms in COVID-19 patients may be attributed to the flexibility they offer in adjusting D-dimer cutoffs based on the clinical pretest probability, which is particularly relevant given the elevated D-dimer levels frequently observed in COVID-19 due to the disease’s proinflammatory and procoagulant states. By integrating the clinical context, these algorithms allow for more nuanced cutoff adjustments, which can help mitigate the risk of false positives often associated with fixed D-dimer cutoffs. This adaptive approach aligns better with the unique hemostatic profile of patients with COVID-19 who frequently present with elevated D-dimer levels even in the absence of PE. However, the trade-off between specificity and sensitivity remains an important consideration. Silva et al. observed that the YEARS algorithm, despite achieving high specificity, resulted in four additional missed PE diagnoses compared to a fixed D-dimer cutoff [19]. Similarly, the PEGeD algorithm demonstrated the highest specificity among the diagnostic algorithms but was associated with five missed diagnoses, underscoring the need to carefully balance the specificity and sensitivity [19]. This balance may be particularly delicate in COVID-19 patients, as suggested by Speksnijder et al., who found higher failure rates in PE exclusion algorithms within this population, indicating that further algorithm adjustments may be necessary [21]. While our findings, alongside those of Vielhauer et al. [36], underscore the potential utility of the YEARS and PEGeD algorithms in patients with COVID-19, further research is warranted. Prospective studies should evaluate the safety and efficacy of these algorithms in larger, more diverse COVID-19 cohorts to validate their diagnostic performance and assess whether additional modifications are required to address the unique risks associated with COVID-19.

It is widely accepted in previous studies that the D-dimer levels are an important predictor of VTE risk in patients with COVID-19. Various studies have reported that different D-dimer cutoff values yield different results in terms of sensitivity and specificity. For example, Kampouri et al. reported that a combination of D-dimers ≥ 3000 ng/mL and a Wells score ≥ 2 provided high specificity in determining VTE risk (91.6% specificity) [37]. This result suggested that the combined assessment of high D-dimer levels and clinical scores reduce unnecessary imaging requests. Similarly, Ventura-Díaz et al. suggested the D-dimer cutoff value for the diagnosis of PE in patients with COVID-19 as 2903 ng/mL, and reported that this value had a sensitivity of 81% [38]. This study suggested that higher D-dimer cutoff values decrease the sensitivity but increase the specificity, contributing to the avoidance of unnecessary investigations. Brem et al. also showed that D-dimer levels were a strong predictor of PE; specifically, levels > 2590 ng/mL were significantly associated with an increased risk of PE [39]. Notwithstanding the above, it was also reported that high cutoff values may lead to an increased number of missed PE cases due to reduced sensitivity. The results of the present study are consistent with the results of previous studies. Increasing the D-dimer cutoff value increased the specificity but decreased the sensitivity; therefore, some PE cases might be missed. A study by Silva et al. similarly reported that the use of high D-dimer cutoff values was not reliable as a strategy to exclude PE because the sensitivity was severely reduced [19]. Increasing the D-dimer cutoff value might be useful to avoid unnecessary investigations but it may increase the risk of overlooking PE cases. In patients with COVID-19, assessing D-dimer levels along with clinical scores and patient characteristics can provide a more reliable and effective diagnostic strategy.

### Limitations

This study has several limitations. As a retrospective, single-center chart review, it relied on recorded notes rather than direct patient observation, which may have impacted the accuracy of clinical assessments. Additionally, the analysis included only patients who underwent CTPA in the emergency department with available D-dimer test results, potentially limiting the generalizability of the findings to a broader population with suspected PE. Approximately 40% of PE-suspected patients who underwent CTPA were excluded due to incomplete data, which may affect the applicability of our findings, as these excluded patients might differ systematically from those included in the analysis. Nevertheless, data completeness was prioritized to ensure the reliable calculation of pretest scores across different algorithms. Future prospective studies with comprehensive data collection are recommended to validate these findings in a more diverse population. Furthermore, this study focused on symptomatic patients who presented to the emergency department with suspected PE and underwent CTPA imaging. Asymptomatic COVID-19 patients, in whom PE might have been detected incidentally, were not included. Consequently, the findings may not fully extend to asymptomatic populations, and future research should consider assessing the diagnostic score performance in both symptomatic and asymptomatic groups. Lastly, this study did not examine the potential effects of COVID-19 vaccination on PE risk or diagnostic performance, which may hold relevance in the postpandemic context. Future research could explore how the COVID-19 vaccination status and timing might interact with PE risk factors and diagnostic algorithms, providing a more comprehensive understanding in both vaccinated and unvaccinated populations.

## 5. Conclusions

Our findings suggest that the COVID-19 pandemic had a limited effect on the diagnostic performance of clinical decision-making algorithms for PE. The accuracy of algorithms incorporating Wells and Geneva scores with varied D-dimer cutoffs appeared generally consistent across COVID-positive and -negative groups. Although the YEARS and PEGeD algorithms showed slight improvements in specificity and accuracy among COVID-19-positive patients, these results should be interpreted with caution. The application of higher D-dimer cutoffs, while potentially increasing specificity, may also elevate the risk of missed PE cases due to reduced sensitivity. Further research, particularly in diverse patient populations, is needed to validate these findings and optimize the use of these algorithms in clinical practice.

## Figures and Tables

**Table 1:** Baseline characteristics, risk factors, and probability scores in patients with and without COVID-19.

Baseline Characteristics	COVID-19 (+) (n = 994)	COVID-19 (−) (n = 429)	*p*-Value
Previous diagnosis of DVT/PE, n (%)	15 (1.5)	10 (2.3)	0.279
Clinical signs of DVT, n (%)	29 (2.9)	29 (6.8)	0.001
Malignancy, n (%)	88 (8.9)	45 (10.5)	0.330
Heart rate > 100 bpm, n (%)	414 (41.6)	186 (43.4)	0.550
Surgery or fracture within 1 month, n (%)	125 (12.6)	61 (14.2)	0.399
Immobilization for 3 days or surgery in 4 weeks, n (%)	125 (12.6)	61 (14.2)	0.399
Unilateral leg edema, n (%)	28 (2.8)	29 (6.8)	<0.001
Unilateral leg pain, n (%)	24 (2.4)	22 (5.1)	0.008
Hemoptysis, n (%)	31 (3.1)	17 (4.0)	0.418
PE as the first diagnosis or equally likely, n (%)	37 (3.7)	8 (1.9)	0.066
Wells score			
Median (IQR)	1.0 (0.0–1.5)	1.5 (0.0–1.5)	0.116 ^&^
Probability of PE according to Wells score			0.034 ^#^
- Low risk, n (%)	942 (94.8)	397 (92.5)	
- Moderate risk, n (%)	40 (4.0)	30 (7.0)	
- High risk, n (%)	12 (1.2)	2 (0.5)	
Geneva score			
Median (IQR)	5.0 (3.0−6.0)	5.0 (4.0−6.0)	0.003 ^&^
Probability of PE according to Geneva score			0.004
- Low risk, n (%)	287 (28.9)	97 (22.6)	
- Moderate risk, n (%)	685 (68.9)	312 (72.7)	
- High risk, n (%)	22 (2.2)	20 (4.7)	
YEARS Algorithm			0.031
0 items, n (%)	910 (91.5)	337 (87.9)	
≥1 item, n (%)	84 (8.5)	52 (12.1)	
PE, n (%)	72 (7.2)	32 (7.5)	0.886

^&^: Mann–Whitney U test, ^#^: Fisher’s Exact test, others chi-square test. PE, pulmonary embolism; DVT, deep vein thrombosis, IQR, interquartile range.

**Table 2:** Prevalence of risk factors in all patients with and without PE.

Risk Factor	PE Patients (n = 104)	Non-PE Patients (n = 1319)	*p*
Age > 65 years, n (%)	60 (57.7)	579 (43.9)	0.006
Previous diagnosis of DVT/PE, n (%)	5 (4.8)	20 (1.5)	0.031 ^#^
Clinical signs of DVT, n (%)	16 (15.4)	42 (3.2)	<0.001 ^#^
Malignancy, n (%)	10 (9.6)	123 (9.3)	0.922
Heart rate > 100 bpm, n (%)	48 (46.2)	552 (41.8)	0.392
Surgery or fracture within 1 month, n (%)	24 (23.1)	162 (12.3)	0.002
Immobilization for 3 days or surgery in 4 weeks, n (%)	24 (23.1)	162 (12.3)	0.002
Unilateral leg edema, n (%)	16 (15.4)	41 (3.1)	<0.001 ^#^
Unilateral leg pain, n (%)	15 (14.4)	31 (2.4)	<0.001 ^#^
Hemoptysis, n (%)	4 (3.8)	44 (3.3)	0.775 ^#^
PE as the first diagnosis or equally likely, n (%)	9 (8.7)	36 (2.7)	0.004 ^#^

^#^: Fisher’s exact test, others chi-square test was used for all comparisons. PE, pulmonary embolism; DVT, deep vein thrombosis.

**Table 3:** Diagnostic performance of algorithms in patients with and without COVID-19.

Algorithm	COVID-19	Sensitivity (%)	Specificity (%)	PPV (%)	NPV (%)	Accuracy (%)	AUC
Wells score + D-dimer 500 ng/mL	(−)	93.75 [85.36–100.00]	5.54 [3.29–7.79]	7.41 [4.86–9.96]	91.62 [80.61–100.00]	12.12 [9.03–15.21]	0.496 [0.392–0.601]
	(+)	97.22 [80.53–100.00]	4.99 [3.58–6.39]	7.40 [5.73–9.07]	95.83 [90.18–100.00]	11.67 [9.67–13.67]	0.511 [0.443–0.579]
*p*-value		0.585 ^#^	0.677	0.996	0.597 ^#^	0.809	0.819 ^%^
Wells score + AADD	(−)	90.63 [80.53–100.00]	8.82 [6.03–11.61]	7.42 [4.82–10.01]	92.11 [83.53–100.00]	14.92 [11.55–18.29]	0.497 [0.393–0.602]
	(+)	97.22 [93.43–100.00]	7.81 [6.08–9.54]	7.61 [5.90–9.32]	97.30 [93.60–100.00]	14.29 [12.11–16.46]	0.525 [0.459–0.592]
*p*-value		0.320 ^#^	0.583	0.904	0.334 ^#^	0.756	0.658 ^%^
Geneva score + D-dimer 500 ng/mL	(−)	93.75 [85.36–100.00]	5.29 [3.09–7.49]	7.39 [4.84–9.93]	91.30 [79.79–100.00]	11.89 [8.83–14.95]	0.495 [0.390–0.600]
	(+)	97.22 [93.43–100.00]	4.99 [3.58–6.39]	7.40 [5.73–9.07]	95.83 [90.18–100.00]	11.67 [9.67–13.67]	0.511 [0.443–0.579]
*p*-value		0.585 ^#^	0.820	0.995	0.591 ^#^	0.907	0.804 ^%^
Geneva score + AADD	(−)	90.63 [80.53–100.00]	8.56 [5.81–11.32]	7.40 [4.81–9.99]	91.89 [83.10–100.00]	14.69 [11.34–18.03]	0.496 [0.391–0.601]
	(+)	97.22 [93.43–100.00]	7.81 [6.08–9.54]	7.61 [5.90–9.32]	97.30 [93.60–100.00]	14.29 [12.11–16.46]	0.525 [0.459–0.592]
*p*-value		0.320 ^#^	0.644	0.895	0.331^#^	0.844	0.644 ^%^

^#^: Fisher’s exact test, ^%^: z test, others chi-square test. Sensitivity, specificity, PPV, NPV, accuracy, and AUC are reported with 95% confidence intervals in brackets. AADD, age-adjusted D-dimer; PPV, positive predictive value; NPV, negative predictive value; AUC, area under the curve.

**Table 4:** Diagnostic performance of YEARS and PEGeD algorithms in patients with and without COVID-19.

Algorithm	COVID-19	Sensitivity (%)	Specificity (%)	PPV (%)	NPV (%)	Accuracy (%)	AUC
YEARS	(−)	84.38 [71.79–96.96]	26.70 [22.34–31.06]	8.49 [5.43–11.55]	95.50 [91.64–99.35]	31.00 [26.63–35.38]	0.555 [0.458–0.653]
	(+)	86.11 [78.12–94.10]	32.75 [29.73–35.78]	9.09 [6.93–11.25]	96.79 [94.84–98.75]	36.62 [33.62–39.61]	0.594 [0.533–0.656]
*p*-value		1.000 ^#^	*0.029*	0.756	0.553 ^#^	*0.041*	0.508 ^%^
PEGeD	(−)	84.38 [71.79–96.96]	27.96 [23.54–32.37]	8.63 [5.52–11.74]	95.69 [91.99–99.39]	32.17 [27.75–36.59]	0.562 [0.465–0.659]
	(+)	86.11 [78.12–94.10]	34.06 [31.00–37.12]	9.25 [7.06–11.45]	96.91 [95.03–98.80]	37.83 [34.81–40.84]	0.601 [0.540–0.662]
*p*-value		1.000 ^#^	*0.030*	0.749	0.555 ^#^	*0.041*	0.503 ^%^

^#^: Fisher’s exact test, ^%^: z test, others, chi-square test. Sensitivity, specificity, PPV, NPV, accuracy, and AUC are reported with 95% confidence intervals in brackets. PPV, positive predictive value; NPV, negative predictive value; AUC, area under the curve.

**Table 5:** Diagnostic performances of different D-dimer cutoffs.

D-Dimer Cutoff (ng/mL)	Sensitivity (%)	Specificity (%)	NPV (%)	+LR	−LR	Correctly Avoided CTPA (n)	Missed PE Diagnosis (n)
500	96.15 [92.46–99.85]	5.08 [3.89–6.26]	94.37 [89.00–99.73]	1.01 [0.89–1.00]	0.76 [0.28–2.04]	67	4
1000	81.73 [74.30–89.16]	33.13 [30.59–35.67]	95.83 [94.00–97.67]	1.22 [1.11–1.35]	0.55 [0.36–0.83]	437	19
2390	52.88 [43.29–62.48]	73.77 [71.39–76.14]	95.21 [93.90–96.52]	2.02 [1.65–2.47]	0.64 [0.52–0.78]	973	49

CTPA, computed tomography pulmonary angiography; PE, pulmonary embolism; NPV, negative predictive value; +LR, positive likelihood ratio; −LR, negative likelihood ratio.

## Data Availability

Data are available in a publicly accessible repository.

## Supplementary Materials

The following supporting information can be downloaded at https://www.mdpi.com/article/10.3390/jcm13237008/s1. The following supplementary tables are available online along with the manuscript: Table S1. Risk assessment for pulmonary embolism according to the Wells and Geneva scores and YEARS algorithm. Table S2. Diagnostic accuracy of the Wells and Geneva scores combined with D-dimer levels, the YEARS algorithm, and the PEGeD algorithm.
