# Supplementary material for: A Comparative Analysis of the Impact of Severe Acute Respiratory Syndrome Coronavirus 2 Infection on the Performance of Clinical Decision-Making Algorithms for Pulmonary Embolism
Source: J Clin Med. 2024 Nov 21;13(23):7008. doi: 10.3390/jcm13237008 (PMC11642087; doi:10.3390/jcm13237008)
Supplement: Supplementary file 1 [file jcm-13-07008-s001.zip › jcm-3292428-supplementary__proof.docx]

**Table S1: Risk Assessment for Pulmonary Embolism According to Wells, Geneva Scores, and YEARS Algorithm**

| Variable | PE Patients  (n = 104) | Non-PE Patients  (n = 1319) | p-value |
| --- | --- | --- | --- |
| Wells Score |  |  |  |
| Median (Q1–Q3) | 1.5 (0.0–3.0) | 1.0 (0.0–1.5) | <0.001^&^ |
| Probability of PE according to Wells score, n () |  |  | <0.001^#^ |
| Low risk | 86 (82.7) | 1253 (95.0) |  |
| Moderate risk | 12 (11.5) | 58 (4.4) |  |
| High risk | 6 (5.8) | 8 (0.6) |  |
| Geneva Score |  |  |  |
| Median (Q1–Q3) | 6.0 (4.0–8.0) | 5.0 (3.0–6.0) | <0.001^&^ |
| Probability of PE according to Geneva score |  |  | <0.001^#^ |
| Low risk, n () | 15 (14.4) | 369 (28.0) |  |
| Moderate risk, n () | 72 (69.2) | 925 (70.1) |  |
| High risk, n () | 17 (16.3) | 25 (1.9) |  |
| YEARS Algorithm |  |  | <0.001 |
| 0 items, n () | 81 (77.9) | 1206 (91.4) |  |
| ≥1 item, n () | 23 (22.1) | 113 (8.6) |  |

^&^: Mann–Whitney U test, ^#^: Fisher’s Exact test, others: chi-square test. PE, Pulmonary Embolism; IQR, Interquartile Range.

**Table S2: Diagnostic Accuracy of Wells, Geneva Scores combined with D-Dimer, YEARS Algorithm, and PEGeD Algorithm**

| Algorithm | Sensitivity  (%) | Specificity  (%) | PPV  (%) | NPV  (%) | PLR | NLR |
| --- | --- | --- | --- | --- | --- | --- |
| Wells score + DD 500 ng/mL | 96.15 [92.46–99.85] | 5.16 [3.96–6.35] | 7.40 [6.01–8.80] | 94.44 [89.15–99.74] | 1.01 [0.97–1.06] | 0.75 [0.28–2.00] |
| Wells score + AADD | 95.19 [91.08–99.30] | 8.11 [6.64–9.59] | 7.55 [6.12–8.98] | 95.54 [91.17–99.36] | 1.04 [0.99–1.08] | 0.59 [0.25–1.42] |
| Geneva score + DD 500 ng/mL | 96.15 [92.46–99.85] | 5.08 [3.89–6.26] | 7.40 [6.00–8.79] | 94.37 [89.00–99.73] | 1.01 [0.97–1.05] | 0.76 [0.28–2.04] |
| Geneva score + AADD | 95.19 [91.08–99.30] | 8.04 [6.57–9.50] | 7.54 [6.12–8.97] | 95.50 [91.64–99.35] | 1.04 [0.99–1.08] | 0.60 [0.25–1.43] |
| YEARS algorithm | 85.58 [78.82–92.33] | 30.93 [28.44–33.43] | 8.90 [7.14–10.66] | 96.45 [94.69–98.22] | 1.24 [1.14–1.35] | 0.47 [0.29–0.75] |
| PEGeD algorithm | 85.58 [78.82–92.33] | 32.22 [29.70–34.74] | 9.05 [7.26–10.85] | 96.59 [94.90–98.29] | 1.26 [1.16–1.38] | 0.45 [0.28–0.72] |

PPV: Positive predictive value, NPV: Negative predictive value, PLR: Positive likelihood ratio, NLR: Negative likelihood ratio, AADD: Age-adjusted D-dimer, DD: D-dimer.
