# Supplementary material for: A Comparative Analysis of the Impact of Severe Acute Respiratory Syndrome Coronavirus 2 Infection on the Performance of Clinical Decision-Making Algorithms for Pulmonary Embolism
Source: J Clin Med. 2024 Nov 21;13(23):7008. doi: 10.3390/jcm13237008 (PMC11642087; doi:10.3390/jcm13237008)
Supplement: Supplementary file 1 [file jcm-13-07008-s001.zip › CertificateOfEditing_KYJDAD-1.pdf]

enago
www.enago.com.tr

voxtab
www.voxtab.com

CERTIFICATE OF EDITING

This is to certify that the paper titled **"Comparative Analysis of the Impact of Severe Acute Respiratory Syndrome Coronavirus 2 Infection on the Performance of Clinical Decision-Making Algorithms for Pulmonary Embolism"** commisioned to us by **Merve Ekşioğlu** has been translated and edited from Turkish to English by **Enago**, a translation brand of Crimson Interactive LLC.

ISO 17100:2015 CERTIFIED
Requirements for Translation Services

ISO 9001 Quality Management
ISO 9001:2015 Certified

ISO/IEC 27001 Information Security Management
ISO/IEC 27001:2013 Certified

Issued by:
Ulatus, Crimso
616 Corporate
Valley Cottage
Phone: 0216 6
06

Disclaimer: The author is free to accept or reject our changes in the document after our translation. However, we do not bear responsibility for revisions made to the document after our translation on **11th November 2024**

English www.enago.com, www.voxtab.com, www.ulatus.com
Japan www.enago.jp, www.voxtab.jp, www.ulatus.jp
Brazil www.enago.com.br
German www.enago.de
Turkey www.enago.com.tr
China www.enago.cn
Taiwan www.enago.tw

About Crimson:
Crimson Interactive LLC. provides English language editing, transcription, and translation services to individuals and corporate customers worldwide.
